# Machine learning surrogate for the leaf PROSPECT-D model and its applications across plant species

**DOI:** 10.1038/s41598-026-53899-1

**Published:** 2026-05-19

**Authors:** Milad Rahimi-Majd, Rudan Xu, Stefan Bauermeister, Zoran Nikoloski

**Affiliations:** 1 Bioinformatics Department, Institute of Biochemistry and Biology, University of Potsdam, 14476 Potsdam, Germany; 2 Systems Biology and Mathematical Modeling Group, Max Planck Institute of Molecular Plant Physiology, 14476 Potsdam, Germany

**Keywords:** Leaf hyperspectral reflectance, PROSPECT-D model, Model transferability, Machine learning, Model inversion, Computational biology and bioinformatics, Plant sciences

## Abstract

Leaf hyperspectral reflectance (HSR) data have gained increasing attention due to their usage in predicting a range of leaf physiological, biochemical, structural, and photosynthetic traits using machine learning (ML) models. The PROSPECT family of models offers a complementary, mechanistic means to estimate leaf traits from HSR data using model inversion. However, a comprehensive evaluation of the accuracy and transferability of the PROSPECT model across a large set of species is hindered by the limited availability of ground truth data sets. Here, we employed a combination of inversion and forward simulation of the PROSPECT-D model across a broad range of species and identified four narrow wavebands linked to environmental effects. We also introduced a novel framework using partial least squares regression to enable the analysis of the transferability of the machine learning models trained base on the PROSPECT-D across species. This analysis revealed trait-specific patterns of transferability for the machine learning surrogate based on the PROSPECT-D forward model. We then extended this analysis to PROSPECT-D inversion using neural networks and developed a fast, accurate deep-learning-based surrogate inversion approach to estimate leaf traits from measured HSR data. Our data-driven framework paves the way for improving the accuracy of PROSPECT and similar mechanistic models.

## Introduction

Leaf hyperspectral reflectance (HSR) is a high-throughput plant phenotyping method that results in rapid, low-cost, and non-destructive measurements. The resulting data have been shown useful in predicting species- and genotype-specific biochemical and physiological^1–7^, structural^8–10^, and photosynthetic^11–16^ traits and providing insights on different plant growth stages^3,17,18^.

HSR data have been used with two types of models, namely data-driven and mechanistic. One important class of data-driven models make use of supervised machine learning (ML) approaches that consider the reflectance values at different wavelengths as predictors and a given leaf trait as a target variable (*e.g.*, see^1^ and references therein for a comprehensive review). Another major class of data-driven models involves vegetation indices that relate narrow wavebands in HSR data to plant traits, at both the canopy and the leaf level^19–21^. The existing data-driven models also rely on other statistical techniques, including: principal component analysis (PCA)^22^, derivative-based methods^23,24^, and Fourier analysis^25^, capturing signals in the HSR data that go beyond regression analyses.

Mechanistic models aim to explain leaf HSR profiles based on physical theories that model the optical interaction of incident rays with leaf pigments and structure regarding light absorption and scattering^26,27^. For instance, PROSPECT^28^, ray tracing^29,30^, stochastic^31^, dorsiventral^32^, and spectral invariants^33^ are typical representatives of mechanistic models. The PROSPECT family of models is currently regarded as one of the most popular and widely used in the remote sensing community. This family of models simulates leaf transmittance and reflectance spectra with high accuracy based on a few leaf structural and biochemical traits. Aside from the original PROSPECT model, the family includes PROSPECT-4 and 5^34^ as well as PROSPECT-D^35^; models like PIOSL^10,36^ and FASPECT^37^ have been developed based on the original PROSPECT framework to further improve accuracy in simulating HSR data. These developments primarily focus on improving how well variations in the simulated spectra across different wavelengths correspond to given values of leaf traits, such as chlorophyll content, as input parameters. Therefore, comprehensive understanding of the performance and errors across different wavelengths is important for future improvements of the PROSPECT modeling framework.

The discrepancy between measured HSR data and those simulated by the PROSPECT family of models has largely focused on model performance at individual wavelengths. This focus was evident in the studies developing this line of models^34,35,38^ as well as in complementary studies that critically analyzed the model performance^39,40^. However, these studies relied on limited sets of matched HSR profiles and leaf traits due to the labor-intensive nature of measuring the leaf biochemical and structural traits required for PROSPECT model simulations^1^.

Another aspect of improving the performance of PROSPECT models relates to the generalization of the model across a diverse set of species. This is driven by the data-based calibration of PROSPECT models based on optical parameters, such as the absorption coefficients of leaf biochemical constraints as well as refractive coefficients^34,35,38^. Since the calibration data is limited to specific species and measurement conditions, a key concern is how well the model is transferable to unseen species and conditions. The direct evaluation of the transferability of the PROSPECT model on unseen species and environmental conditions is not facile due to the lack of matched experimental data on HSR and leaf traits which can also be predicted with the PROSPECT model.

The PROSPECT models simulate HSR profiles based on leaf traits as parameters. The importance of the PROSPECT models in applications is the ability to invert them, thereby allowing the estimation of leaf traits from observed HSR profiles^40–42^. This inversion process presents another major challenge not only for PROSPECT, but for all mechanistic models, due to the iterative nature of inversion methods. In particular, the model inversion is often an ill-posed problem^43^ (*i.e.* resulting in non-unique leaf traits associated with the same HSR data); further, the computational costs for inversion are significantly higher than those of the forward simulation, highlighting the need for more efficient surrogate modeling techniques.

Here, we provide a data-driven framework that addresses the three challenges for further improvements of the PROSPECT family of models. Our framework offers: (*i*) an extensive error analysis of PROSPECT model on a broad range of data sets across multiple species, (*ii*) the data-driven framework for transferability analysis of the ML-based models trained on top of the PROSPECT-D forward and inversion modes across diverse species data sets, and (*iii*) a fast, deep-learning-powered, transferable surrogate for inversion that accurately retrieves PROSPECT leaf traits from leaf measured HSR data. In this regard, our analysis characterizes both general and species-specific error trends in the PROSPECT model across wavelengths, linking these patterns to environmental and genetic factors. We also offer an explanation for the transferability patterns of the ML models trained on the PROSPECT forward mode across different data sets using partial least squares regression (PLSR) models. Finally, we explore the corresponding transferability patterns for the models trained based on the PROSPECT inversion mode and, based on this analysis, develop a neural network (NN) framework to efficiently invert PROSPECT-like models.

## Methods

### Description of the experimental data sets

To provide a comprehensive analysis of the performance of the PROSPECT-D model, we used a large set of previously published spectral data sets. In this regard, we conducted our analysis on nine data sets comprising a total of 7548 leaf HSR samples, as follows:

- A recently published maize data set, including 3749 samples of leaf HSR profiles collected from a field-grown maize Multiparent Advanced Generation Inter-Cross (MAGIC) population. The data were gathered in 2021 and 2022 at the National Institute of Agricultural Botany (NIAB) in Cambridge, UK^25,44^. We refer to this data set as UCAM Maize.
- Four maize (with 1734 samples), sorghum (with 949 samples), camelina (with 96 samples), and soybean (with 126 samples) data sets, collected from a series of 11 field and greenhouse experiments conducted between 2018 and 2020, associated with the University of Nebraska–Lincoln^5^. We refer to these data sets as UNL Maize, UNL Sorghum, UNL Camelina, and UNL Soybean, respectively.
- A data set for tropical tree species, *i.e.*, *Erisma uncinatum*, including 72 HSR samples, collected in 2012 at the K67 eddy covariance tower site in the Tapajós National Forest near Santarém, Brazil^45^. We refer to this data set as Tropical.
- A data set including 184 HSR samples collected from eight crop species: *Solanum lycopersicum var. lycopersicum*, *Cucumis sativus*, *Cucurbita pepo*, *Glycine max*, *Phaseolus vulgaris*, *Ocimum basilicum*, *Helianthus annuus*, and Poplar cuttings (*Populus deltoides* Bartr. $\times$ *Populus nigra* L.) measured on fully expanded leaves in a glasshouse environment^46^. Following the terminology of a previous study^1^, we refer to this data set as Eudicot.
- Finally, two frequently used data sets, LOPEX (with 330 samples)^47^ and ANGERS (with 308 samples)^35,41^, that include samples from a diverse set of woody and herbaceous species. Each data set comprises about 50 species.

To ensure a consistent wavelength range across all data sets, we adopted the range available in the ANGERS data set, *i.e.*, 400–$2450~\textrm{nm}$, for all HSR samples analyzed. A summary of all datasets indicating sample size, vegetation functional types, the availability of measured parameters, and geographic information is available in Table 1.

**Table 1:** Summary of hyperspectral reflectance data sets used in this study.

Dataset	Sample size	Vegetation type	Measured parameters’ availability	Experiment reference location
UCAM Maize	3749	Crop (Maize)	^25,44^	Cambridge, UK
UNL Maize	1734	Crop (Maize)	^5^	Nebraska, USA
UNL Sorghum	949	Crop (Sorghum)	^5^	Nebraska, USA
UNL Camelina	96	Crop (Camelina)	^5^	Nebraska, USA
UNL Soybean	126	Crop (Soybean)	^5^	Nebraska, USA
Tropical	72	Tropical tree (*E. uncinatum*)	^45^	Santarém, Brazil
Eudicot	184	Multiple crop species	^46^	Upton, NY, USA
LOPEX	330	Woody and herbaceous species	^47^	Ispra, Italy
ANGERS	308	Woody and herbaceous species	^41^	Angers, France
Total	7548			

### PROSPECT-D model implementation

In this study, we used the latest version of the PROSPECT model, namely PROSPECT-D, which simulates leaf directional-hemispherical reflectance and transmittance based on a set of biochemical constituents and a structural parameter^35^. These parameters are: leaf chlorophyll content (*CH*), carotenoid content (*CAR*), anthocyanin content (*ANT*), equivalent water thickness (*EWT*), leaf dry mass per area (*LMA*), leaf brown pigment (*Brown*), and the leaf structure parameter (*N*). In all our analyses, we excluded the brown pigment parameter and relied only on the remaining six parameters. This was based on the fact that brown pigments are specific to late senescent stages and are absent in juvenile, mature, and early senescent leaves^48^.

Although the standard inversion mode of the PROSPECT model relies on both reflectance and transmittance data sets, acquiring both experimentally is often laborious and time-consuming. Consequently, it is common practice to use solely reflectance data, which are easier to measure^40^. Following this approach, we excluded leaf transmittance data and performed our analysis only on reflectance data sets.

To estimate the PROSPECT parameters from a given HSR profile, we used the inversion of the PROSPECT-D model, which provides the input parameters yielding the best fit between the simulated and target HSR profiles^35,42^. As a result, we estimated six structural and biochemical traits of the leaf for each HSR profile: the estimated chlorophyll content ($CHL_{inv}$), estimated carotenoid content ($CAR_{inv}$), estimated anthocyanin content ($ANT_{inv}$), estimated equivalent water thickness ($EWT_{inv}$), estimated leaf dry mass per area ($LMA_{inv}$), and the estimated number of mesophyll layers (leaf structure parameter) ($N_{inv}$).

All computations in both the forward and inversion modes of PROSPECT-D were conducted using the existing R package *prospect*, version 1.7.2^48^.

### Synthetic data generation

For each experimental HSR data set, we generated a sequence of synthetic data sets based on the PROSPECT-D forward and inversion models. Specifically, for each measured HSR data set ($HSR_{meas}$), we first estimated the set of PROSPECT traits, $P_{inv}$, *i.e.*, $N_{inv}$, $CHL_{inv}$, $EWT_{inv}$, $LMA_{inv}$, $CAR_{inv}$, and $ANT_{inv}$, using the inversion mode of the PROSPECT-D model. Next, we used these estimated parameters in the forward mode of the PROSPECT-D model to generate a corresponding simulated HSR data set ($HSR_{sim}$) for each $HSR_{meas}$.

**Fig. 1:**
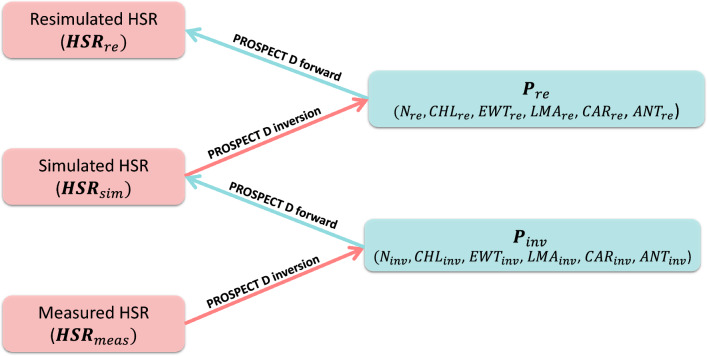
Workflow of synthetic data generation. The schematic process of generating synthetic HSR data sets, $HSR_{sim}$ and $HSR_{re}$, together with the inversion parameters, $P_{inv}$ and $P_{re}$, based on the measured hyperspectral reflectance data, $HSR_{meas}$, using the PROSPECT-D forward and inversion models.

We then repeated this process for the $HSR_{sim}$ data sets, resulting in a re-estimated set of PROSPECT input parameters, $P_{re}$–namely, $N_{re}$, $CHL_{re}$, $EWT_{re}$, $LMA_{re}$, $CAR_{re}$, and $ANT_{re}$–and a corresponding re-simulated HSR data set ($HSR_{re}$) for each pair of $HSR_{meas}$ and $HSR_{sim}$. The schematic workflow of the data generation process is shown in Fig. 1.

### Estimation of broad-sense heritability

The maize metadata used for heritability analysis was collected over two years (2021 and 2022), with 306 of the 326 lines common to both years. Each line in the data set has 1–2 replicates per year, corresponding to distinct blocks in the alpha-lattice design. For each replicate, HSR measurements were recorded in 1–3 repeats from individual plants.

Heritability estimates for this data set followed the approach used in a recent study^25^. Treating each measurement year as a distinct environment, we applied the standard model of broad-sense heritability^49–51^, given by:

1 $$\begin{aligned} \sigma ^2_{p_{\bar{n}}} = \sigma ^2_g + \dfrac{\sigma ^2_{ge}}{n_{e}} + \dfrac{\sigma ^2_{\epsilon }}{n_{e}n_{r}}, \end{aligned}$$

where $\sigma ^2_g$, $\sigma ^2_{ge}$, and $\sigma ^2_{\epsilon }$ represent the variance components due to genotype, genotype-by-environment interaction, and residual error, respectively. The variables $n_{e}$ and $n_{r}$ denote the number of environments and replicates per genotype, respectively^52^. All calculations of heritability were performed using the R source codes from the META-R software^52^.

### Partial least-squares regression (PLSR) modeling of HSR data

In the context of developing ML models with HSR data, the PLSR model has been extensively applied^1,53^. This is primarily due to the high multicollinearity and the large number of predictors (*i.e.*, reflectance values at different wavelengths), for which PLSR is widely regarded as an effective method^54^. The model achieves dimensionality reduction by transforming the original predictors into a smaller set of latent variables that maximize covariance with the response variables^55^.

Here, we used PLSR model to predict PROSPECT inversion traits ($P_{inv}$) based on HSR profiles, while the models were trained and tested on different data sets. To this end, for all PLSR models, we used 30 latent variables which demonstrated a high performance for training models of predicting leaf traits based on HSR data in previous work (*e.g.*, see^5,25^). All PLSR models were implemented using the Python package Scikit-learn^56^.

### Neural network model

In addition to the PLSR models, we also employed multi-layer feed-forward neural network (NN) models as they are well-suited for capturing non-linear and complex relationships. These models are based on an architecture inspired by biological neural networks, where each node, similar to a biological neuron receives input, processes it through an activation function, and transmits the output to other nodes. This process enables the network to learn the patterns and predict and classify the output variables by iteratively adjusting the connection weights through the back-propagation of the learning errors^57^.

In all our analyses, we developed a neural network model with four hidden layers, each using Rectified Linear Unit (ReLU) activation. To enhance generalization and reduce overfitting, we applied *L*2 regularization with a small weight decay parameter (explained below). Additionally, we employed a learning rate scheduler that decreased the learning rate every 10 steps using a decay factor of 0.95. Using this framework, we aimed to develop models that provide optimal performance in estimating each individual PROSPECT inversion trait, *i.e.*, $N_{inv}$, $CHL_{inv}$, $EWT_{inv}$, $LMA_{inv}$, $CAR_{inv}$, and $ANT_{inv}$, based on the measured reflectance profiles, $HSR_{meas}$. To this end, we conducted extensive hyperparameter tuning based on the LOPEX and ANGERS data sets, which exhibited the greatest species diversity among the data sets analyzed (see Sect. "Description of the experimental data sets"). In this process, we conducted a manual grid search by combining multiple configurations of hidden layers–(1024, 512, 256, and 128), (512, 256, 128, and 64), continuing down to (64, 32, 16, and 8)–with a wide range of learning rate ($\alpha$) and *L*2 regularization weight decay (*w*) values, selected from the set 0.5, 0.1, 0.05, 0.01, 0.005, 0.001, 0.0005, 0.0001, 0.00005, and 0.00001. This grid search enabled us to identify the optimal set of hyperparameters for each individual inversion trait and data set. Based on this evaluation, we fixed the sizes of the hidden layers to 512, 256, 128, and 64 for all models. In the same way, we established fixed learning rates for different traits as: $\alpha =0.0005$ for *N*, *CHL*, *EWT*, and *LMA*; and $\alpha =0.001$ for *CAR* and *ANT*. Similarly, we fixed the weight decay values as: $w=0.001$ for *N*, *CHL*, and *EWT*; $w=0.005$ for *LMA*; $w=0.00005$ for *CAR*; and $w=0.00001$ for *ANT*.

All models were trained using a batch size of 128 for 500 epochs ensuring sufficient data exposure to the model and stable convergence across different configurations (see Fig. S1). All models were implemented using the Python package PyTorch 2.5.1^58^.

## Results

### Accuracy of PROSPECT model across wavelengths

Although PROSPECT models are highly efficient in simulating the leaf hyperspectral reflectance (HSR) profile of plant species (see Sect. "Introduction"), discrepancies between the simulated and measured profiles remain unexplained. To address this question, ideally, in addition to measured HSR data, the full set of input traits required by the PROSPECT model (*e.g.*, $N_{inv}$ and $CHL_{inv}$) should also be measured to generate the corresponding simulated HSR profiles. However, these biochemical and structural traits are difficult to measure under the same conditions and on the same set of leaves. As a result, most currently available data sets do not include matched data for these traits along with the corresponding HSR profiles.

Here, we developed a simulation approach for measured hyperspectral reflectance data by combining the PROSPECT-D inversion and forward models. This approach allowed us to generate equivalent simulated data ($HSR_{sim}$) for each measured hyperspectral reflectance data set ($HSR_{meas}$) we analyzed (see Sect. "Synthetic data generation").

To identify potential biases introduced by the PROSPECT model, we calculated the mean relative error, ${\bar{\delta }}_{meas,sim}$, and the Pearson correlation coefficient, $r_{meas,sim}$, between the samples of $HSR_{meas}$ and $HSR_{sim}$ across wavelengths. The mean relative error represents the average magnitude of the simulated reflectance error relative to the measured reflectance, averaged over samples at each wavelength, defined as:

2 $$\begin{aligned} {\bar{\delta }}_{meas,sim}(\lambda ) =\frac{1}{n} \sum _{i=1}^{n} \frac{|HSR_{sim}(\lambda ,i) - HSR_{meas}(\lambda ,i)|}{|HSR_{meas}(\lambda ,i)|}, \end{aligned}$$

where *n* is the number of $HSR_{meas}$ ($HSR_{sim}$) samples. The Pearson correlation, on the other hand, serves as the predictability score of the PROSPECT model for the reflectance of the leaf, under incident light at a given wavelength, regardless of the error magnitude.

**Fig. 2:**
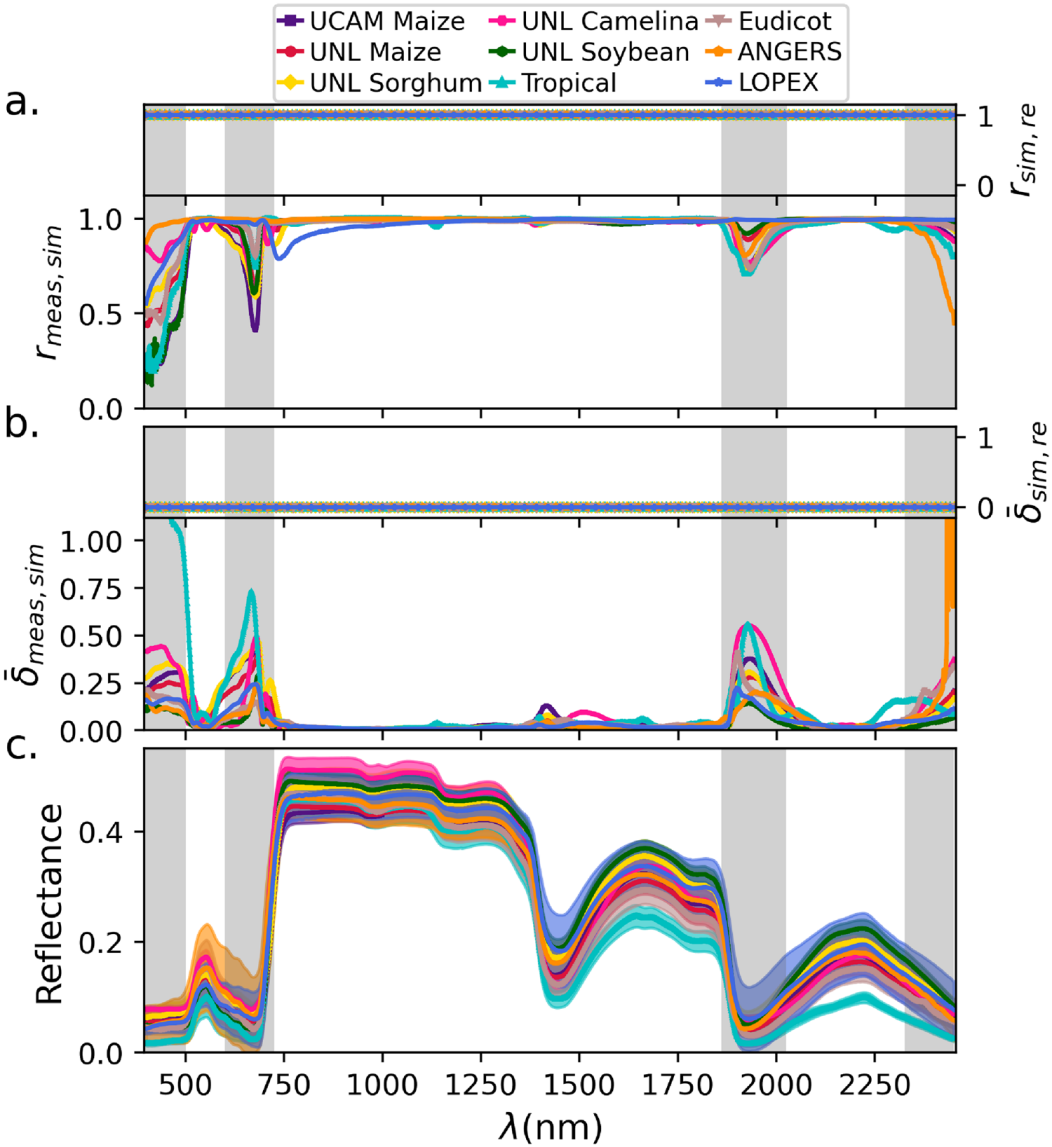
Accuracy of the PROSPECT model across wavelengths. (**a**) Pearson correlation ($r_{meas,sim}$) and (**b**) average relative error (${\bar{\delta }}_{meas,sim}$) between measured ($HSR_{meas}$) and simulated ($HSR_{sim}$) leaf hyperspectral reflectance across wavelengths from 400 to $2450\ \textrm{nm}$, for data sets of nine species: UCAM Maize, UNL Maize, UNL Sorghum, UNL Camelina, UNL Soybean, ANGERS, LOPEX, Eudicot, and Tropical (see Description of the experimental data sets). To enhance the visual representation of the variation, the upper bound of the y-axis in panel (b) was set to 1. Insets in panels (a) and (b) show the corresponding values–$r_{sim,re}$ and ${\bar{\delta }}_{sim,re}$–between the simulated ($HSR_{sim}$) and re-simulated ($HSR_{re}$) reflectance for the same data sets. (**c**) The average of $HSR_{meas}$ samples across wavelengths for the analyzed data sets, with shaded regions in matching colors representing the corresponding standard deviations.

We found that both of our measures demonstrated excellent performance for the PROSPECT model in regenerating all nine analyzed HSR data sets across all wavelengths (Fig. 2a,b). The exception to this finding were four specific wavebands, covering approximately the following ranges: $400-500~nm$, $600-725~nm$, $1860-2025~nm$, and $2325-2450~nm$ (Fig. 2a,b). These wavebands, in fact, closely aligned with the ranges where reflectance values were notably small, tending to zero (Fig. 2c).

Next, we asked whether these errors result from the iterative optimization in the inversion process of the PROSPECT model (*e.g.*, see^43^) or they originate from the mechanistic aspects of the forward model itself. To this end, we computed the same statistics between the simulated ($HSR_{sim}$) and re-simulated ($HSR_{re}$) hyperspectral reflectance data sets. Our results showed no observable error at any wavelength between the simulated and re-simulated profiles (see inlay panels of Fig. 2a,b). This finding indicated that the wavebands that cannot be accurately simulated are associated with mechanistic biases of the PROSPECT model in the forward mode; they also highlight the perfect accuracy of the inversion model in predicting the structural and biochemical traits (*i.e.*, $P_{re}$) from the HSR profiles given to the mechanistic model as input.

In addition to the two previously mentioned accuracy measures, we also assessed the root mean square error (RMSE) between the profiles from $HSR_{meas}$ and $HSR_{sim}$ ($RMSE_{meas,sim}$), as this metric has been frequently used in previous studies for error assessment^34,35,38–40^. This metric also revealed notable errors in the previously discussed wavebands. However, unlike the other metrics, some generally smaller, yet visible, errors were also observed at other wavelengths, particularly in the range $1100-1700~nm$ (Fig. S2a). Nevertheless, since our primary aim in the error analysis was to assess the transferability of PROSPECT model predictions across data sets and species, we focused on Pearson correlation and average relative error, as these metrics emphasize similarity in overall trends and are therefore more indicative of the model’s generalization potential.

### Environmental effects explain the errors of the PROSPECT-D model

As we indicated in the previous section, the wavelength ranges with considerable errors between $HSR_{meas}$ and $HSR_{sim}$ corresponded to the ranges with low reflectance values. This observation raised the question whether these ranges also exhibit lower measurement accuracy. To address this question we computed the broad-sense heritability ($H^2$) of the measured reflectance at each wavelength, treating each as an individual trait. To this end, we used the UCAM Maize data set, which included 3749 samples from 326 unique lines (Sect. "Description of the experimental data sets").

Broad-sense heritability is defined as the proportion of phenotypic variation attributable to genotypic variance^59^:

3 $$\begin{aligned} H^2 = \sigma ^2_g / \sigma ^2_p, \end{aligned}$$

where the boundary values $H^2 = 0$ and $H^2 = 1$ represent cases in which the trait is entirely determined by environmental factors or genetic components, respectively. In this context, we considered the measurement years (*i.e.*, 2021 and 2022) of the HSR profiles as the environmental effect^51^ (see Sect. "Estimation of broad-sense heritability").

**Fig. 3:**
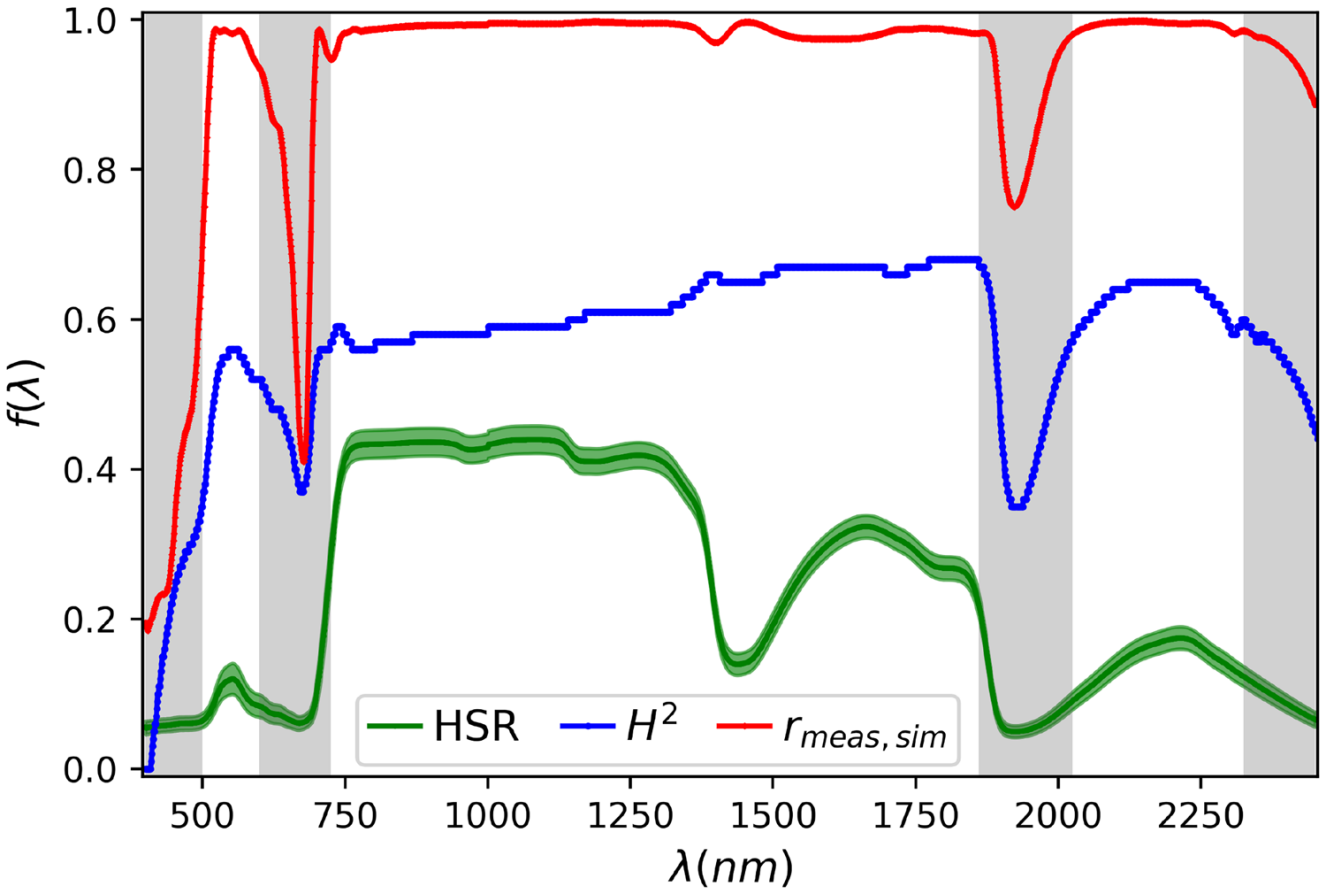
Broad-sense heritability of measured hyperspectral reflectance ($HSR_{meas}$) profile in maize. The profiles of the average of $HSR_{meas}$ samples (with a shaded region representing the corresponding standard deviations), the Pearson correlation ($r_{meas,sim}$) between $HSR_{meas}$ and simulated ($HSR_{sim}$) leaf hyperspectral reflectance, and the broad-sense heritability ($H^2$) across wavelengths for the UCAM Maize data set.

In this analysis, we observed that heritability values ranged from $H^2 = 0$ for wavelengths between $400-408~nm$, to $H^2 = 0.68$ for wavelengths between $1773-1860~nm$. Interestingly, broad-sense heritability exhibited similar trends to $r_{meas,sim}$ across wavelengths, with wavelengths showing lower accuracy and lower reflectance values generally corresponding to smaller $H^2$ values. This clearly indicated that the errors in the PROSPECT model are associated with the ranges of more pronounced environmental effects (Fig. 3).

### The PROSPECT-D forward model shows excellent transferability across plant species

Despite the considerable potential of HSR data to predict leaf traits^1^, machine learning models trained on HSR data suffer from poor transferability across different environments and species^5^. In this line, we asked whether the ML models trained on the simulations from the PROSPECT model, in forward mode, are transferable across different data sets. More specifically, we investigated the extent to which the relationship between $P_{inv}$ and $HSR_{sim}$ is transferable across different species and data sets, when encoding those relationships as independent ML models. To this end, we trained partial least squares regression (PLSR) models (see Sect. "Partial least-squares regression (PLSR) modeling of HSR data") with the reflectance values at different wavelengths of $HSR_{sim}$ used as model predictors and each individual trait from $P_{inv}$ employed as the target (response) variable. A PLSR model was trained on each data set, individually, and the model transferability was tested on the remaining data sets. The performance of the models on the test data sets was evaluated using the Pearson correlation coefficient (*r*) and the coefficient of determination ($R^2$) between the observed and predicted values for the target variables.

**Fig. 4:**
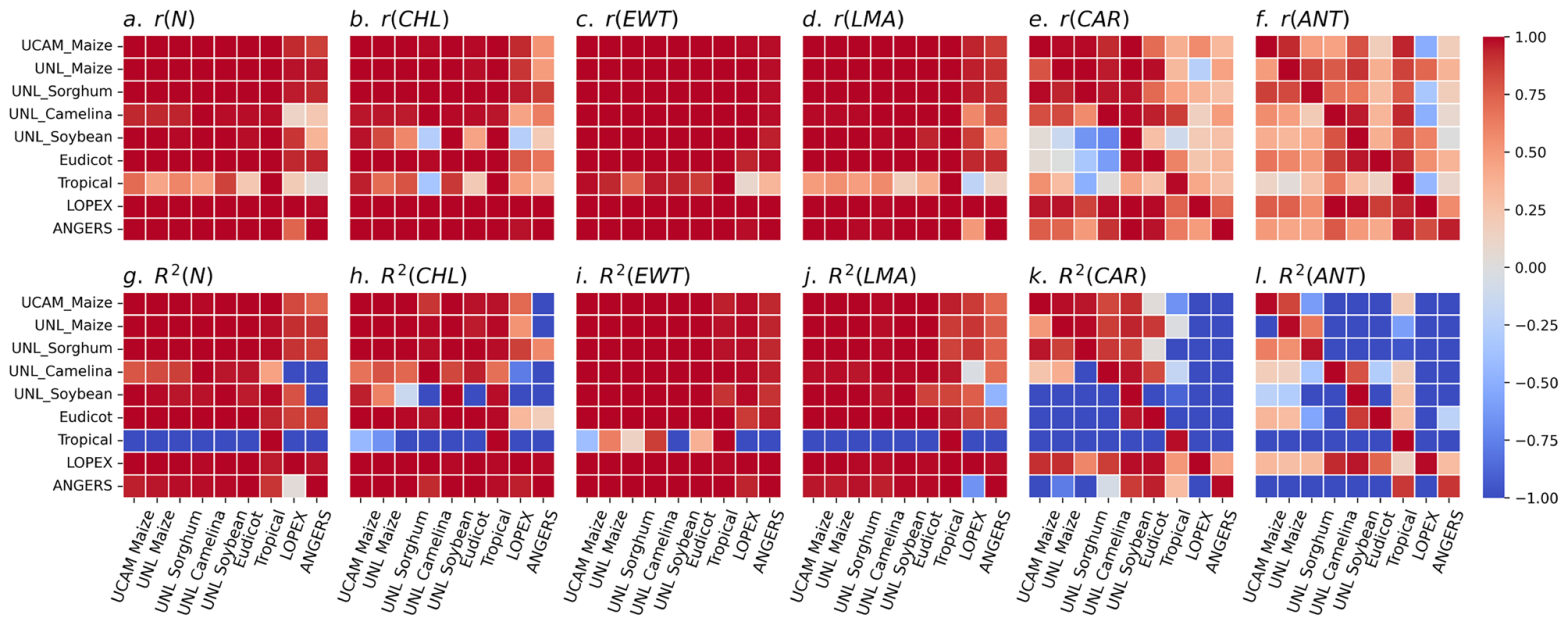
Transferability of the PROSPECT-D model in the forward mode based on partial least squares regression (PLSR) models across plant species. (**a**–**f**) The heatmap plots of pairwise Pearson correlation (*r*) predictability of prospect inversion parameters, $P_{inv}$, *i.e.*, leaf structure parameter ($N_{inv}$), chlorophyll content ($CHL_{inv}$), equivalent water thickness ($EWT_{inv}$), leaf mass per area ($LMA_{inv}$), carotenoid content ($CAR_{inv}$), and anthocyanin content ($ANT_{inv}$), respectively, through the trained models on their corresponding simulated hyperspectral reflectance ($HSR_{sim}$) profiles. The rows and columns of the heatmap plots indicate the train and test data sets, respectively. (**g**–**l**) The same as with (a–f), respectively, with the predictability score corresponding to the coefficient of variation $R^2$. To retain the same scale, the values of $R^2<-1$ are denoted by $-1$. All the trained models are based on PLSR with 30 components.

For four traits, namely, $N_{inv}$, $CHL_{inv}$, $EWT_{inv}$, and $LMA_{inv}$, we observed excellent predictability values (*i.e.*, $r \rightarrow 1$ and $R^2 \rightarrow 1$) in the majority of cases (Fig. 4a–d,g–j). The exceptions were mostly found in cases where ANGERS or LOPEX were used as test data sets. On the other hand, models trained on these two data sets achieved high accuracy on all other data sets. This clearly highlights the impact of greater variation in the predictors and target variables in the ANGERS and LOPEX data sets relative to the others, due to their higher species diversity. As another special case, models trained on the Tropical data set showed notably weak predictability performance for the four traits when tested on the other data sets. In contrast, models trained on the other data sets yielded high predictability scores for these traits on the Tropical used as a test data set (Fig. 4a–d,g–j).

For $CAR_{inv}$, we generally observed weak transferability between data sets, except in a few cases. These included models trained and tested on the UCAM Maize, UNL Maize, and UNL Sorghum data sets, as well as models trained on the LOPEX data set and tested on the others (Fig. 4e,k). Finally, $ANT_{inv}$ showed a similar trend to $CAR_{inv}$, but with fewer high-performing models, particularly in terms of $R^2$ (Fig. 4f,l).

These general patterns show that the PLSR models trained on the PROSPECT-D forward model is highly transferable between species for the four traits $N_{inv}$, $CHL_{inv}$, $EWT_{inv}$, and $LMA_{inv}$, while this transferability is weak for $CAR_{inv}$ and $ANT_{inv}$.

### Sensitivity analysis explains the transferability patterns of the PROSPECT-D model in the forward mode

Investigating the factors underpinning the transferability of the PLSR models trained based on the PROSPECT-D forward mode across different data sets could be relevant for further assessment of leaf radiative transfer mechanistic models, in addition to their predictive applications. In this context, we performed a sensitivity analysis with the aim of exploring the effect of each individual trait of $P_{inv}$ on the simulated HSR profiles. To this end, for each data set, we applied the PROSPECT-D forward model on $P_{inv}$ parameters (*i.e.*, $N_{inv}$, $CHL_{inv}$, $EWT_{inv}$, $LMA_{inv}$, $CAR_{inv}$, and $ANT_{inv}$) such that, for simulating each profile in $HSR_{sim}$, one of the inversion parameters retained its actual value, while the other five traits were held constant at their average values across samples of the corresponding data set. We then made use of the coefficient of variation (CV), defined as the ratio of the standard deviation ($\sigma$) to the mean ($\mu$) of the samples (CV=$\sigma$/$\mu$), as a sensitivity measure of the HSR to the modulated parameter across the different wavelengths. To complement this analysis, we also examined the distributions of the $P_{inv}$ parameters for each data set, which capture the statistics of the variation for each trait.

**Fig. 5:**
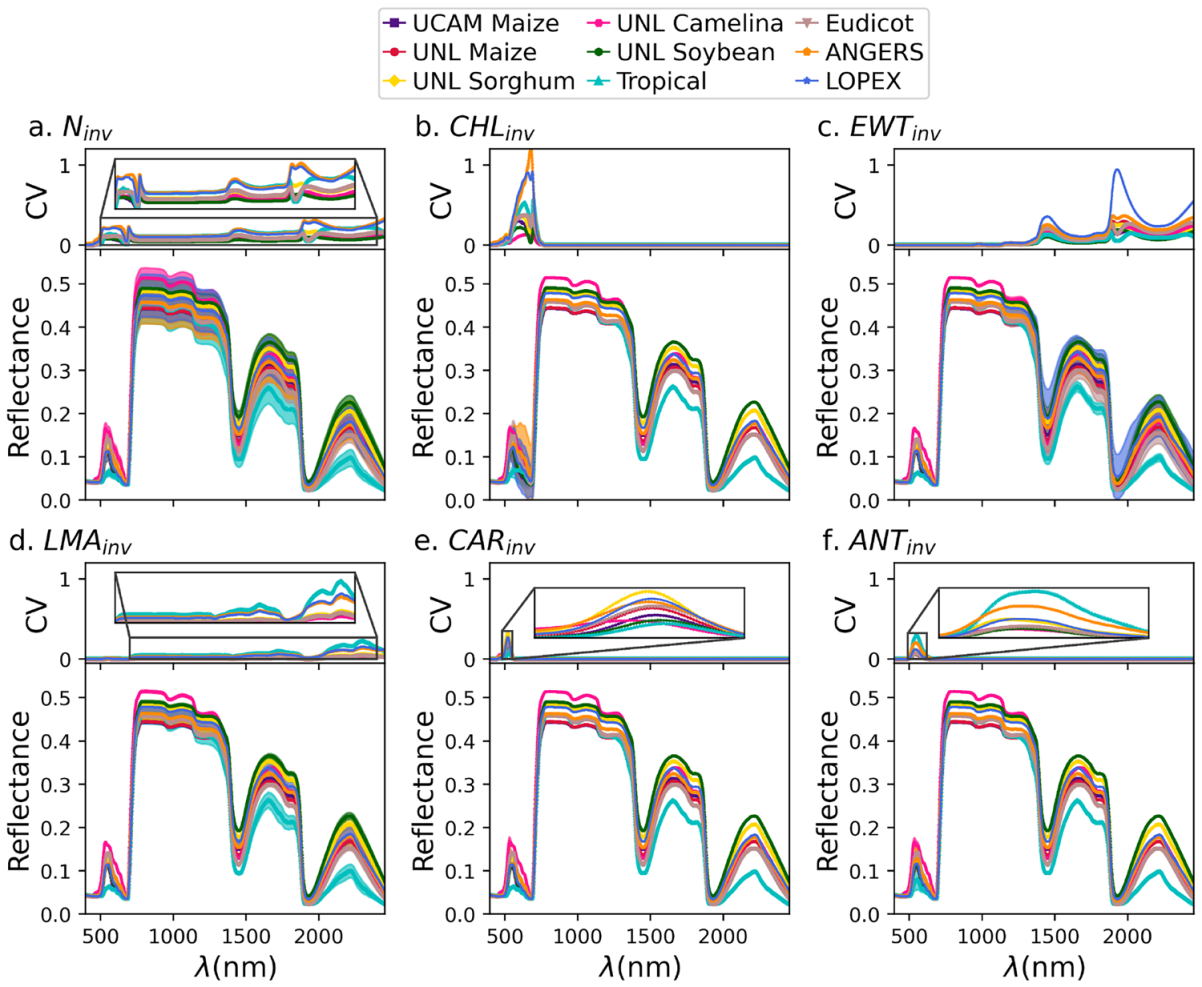
Sensitivity analysis of PROSPECT forward model across wavelengths. The average simulated hyperspectral reflectance ($HSR_{sim}$) across wavelengths, based on variations in the following parameters, (**a**) leaf structure parameter ($N_{inv}$), (**b**) chlorophyll content ($CHL_{inv}$), (**c**) equivalent water thickness ($EWT_{inv}$), (**d**) leaf mass per area ($LMA_{inv}$), (**e**) carotenoid content ($CAR_{inv}$), and (**f**) anthocyanin content ($ANT_{inv}$) for nine species data sets: UCAM Maize, UNL Maize, UNL Sorghum, UNL Camelina, UNL Soybean, ANGERS, LOPEX, Eudicot, and Tropical: In the simulation of each profile, one of the inversion parameters retained its actual value, while the other five traits were held constant, at their average values across samples from their corresponding data set. The shaded regions in matching colors represent the corresponding standard deviations. Inset panels show the corresponding coefficient of variation (CV) values of $HSR_{sim}$ at each wavelength.

Based on the sensitivity analysis, we observed that each trait affects specific wavebands of the HSR profile (Fig. 5). This is indeed in agreement with the mechanistic theory and calibration process of the PROSPECT model (*e.g.*, see^34^), as well as sensitivity analyses from previous studies^39,60^. Our observations indicated that the magnitude of CV values (*i.e.*, sensitivity intensity) varies significantly across different data sets (Fig. 5). This variations mechanistically originate from the average values of the five fixed traits and, more importantly, from the distribution of values of the varying trait on which the sensitivity analysis was performed for each data set (Fig. S3).

From a statistical perspective, achieving a transferable model for a trait between data sets requires that their sensitive wavebands at least partially align. Importantly, for two low-performance traits, $CAR_{inv}$ and $ANT_{inv}$, we observed that not only is the sensitivity very low, but the wavelength domain over which different data sets align is also considerably narrower (Fig. 5e,f). At the same time, the distributions of these two traits, especially $ANT_{inv}$, exhibited long tails across different data sets, compared to the distributions of other traits (Fig. S3e,f). As a result, these patterns suggested a high potential for parameter redundancy or an identifiability problem^61^ for these two traits, in which different input parameter combinations do not lead to significantly different outcomes. This was in line with the weak transferability performance of $CAR_{inv}$ and $ANT_{inv}$, as described above (Fig. 4e,f,k,l).

For the three traits, $N_{inv}$, $EWT_{inv}$, and $LMA_{inv}$, we observed highly extended sensitivity patterns across the wavebands of different data sets (Fig. 5a,c,d). In addition, the sensitive waveband for $CHL_{inv}$ was narrower, but it exhibited a high intensity across different data sets. These patterns for the four traits were, in turn, consistent with the high transferability observed in the trained models for them (Fig. 4a–d,g–j).

The weak (high) predictive performance of the PLSR models trained (tested) on the Tropical data set (Fig. 4) is another major pattern that can be explained based on the sensitivity analysis. In this regard, we observed that the distributions of the traits from the PROSPECT-D inversion was considerably different from those of other data sets (Fig. S3). More specifically, this data set showed generally bimodal distributions, which was significantly different from those of the other five inversion traits (Fig. S3). This pattern was in line with the observation of a distinct range of HSR profiles for this data set, where reflectance values were generally lower in $HSR_{meas}$ (Fig. 2c) and consequently in $HSR_{sim}$ (Fig. 5) profiles. On the other hand, only a minor overlap was found between the sensitivity ranges of this data set and those of the others across the sensitive wavebands (see the shaded areas of the reflectance profiles in Fig. 5), suggesting a domain shift^62^ between the feature spaces of the mentioned data sets. Meanwhile, the high performance of the models tested on this data set (but trained on others) is likely due to the fact that transferring from models trained on data sets with broader coverage is generally easier than from those trained on more limited data, with small coverage. This is in line with the Tropical HSR data that were gathered from a single species, *i.e.*, *Erisma uncinatum*, with the smallest sample number among all the data sets analyzed (see Sect. "Description of the experimental data sets").

### Neural network models robustly maintain the transferability of PROSPECT inversion model across plant species

Aside from understanding the transferability of the ML models trained based on the PROSPECT-D forward mode, it is also relevant to investigate the transferability of those models trained on the PROSPECT inversion mode, *i.e.*, the relationship between $HSR_{meas}$ and $P_{inv}$, across plant species. This scenario is particularly important for real-world analyses of HSR data, as it directly involves the measured hyperspectral reflectance, $HSR_{meas}$, data. In this regard, we first trained PLSR models between $HSR_{meas}$ and $P_{inv}$ using the same configurations as in the $HSR_{sim}$-$P_{inv}$ scenarios described in Sect. "The PROSPECT-D forward model shows excellent transferability across plant species". These analyses, in contrast to the previous scenarios, showed notably weaker predictive performance across species, particularly in terms of $R^2$ values (Fig. S4). This weaker performance was especially evident in models trained on the diverse data sets LOPEX and ANGERS (Fig. S4). To investigate if this weak performance was due to the application of PLSR, a linear regression model, we developed fully connected feedforward neural network (NN) models with four hidden layers (see Sect. "Neural network model"). To this end, based on the performance of preliminary tested models, six NN models were trained for each training data set, each corresponding to one target variable, *i.e.*, $N_{inv}$, $CHL_{inv}$, $EWT_{inv}$, $LMA_{inv}$, $CAR_{inv}$, and $ANT_{inv}$ (see Sect. "Neural network model"). We then tested the trained NN models on all remaining data sets.

In general, the results of the NN models showed comparably similar predictability trends for the $HSR_{meas}$-$P_{inv}$ scenarios to those of the $HSR_{sim}$-$P_{inv}$ scenarios obtained with the PLSR models (Fig. S5 and Fig. 4). This finding not only demonstrates the strong transferability of the ML models based on the PROSPECT-D inversion mode across different plant species, but also highlights the potential of NN models to provide data-based surrogate approaches for inverting the PROSPECT mechanistic model (see below).

### Feature importance of neural network models explains the high transferability of PROSPECT inversion models

The notable transferability of the four traits, namely: $N_{inv}$, $CHL_{inv}$, $EWT_{inv}$, and $LMA_{inv}$, across plant species and data sets in $HSR_{sim}$-$P_{inv}$ scenarios is primarily attributed to the mechanistic nature of the PROSPECT-D model and its calibration process, *e.g.*, based on the absorption coefficients of leaf biochemical contents (see^34,35,38^). However, the transferability in $HSR_{meas}$-$P_{inv}$ scenarios depends also on the measurement accuracy of HSR data and the inversion accuracy of the PROSPECT model, in addition to the factors mentioned above. Since the $HSR_{meas}$ profiles exhibit a robust pattern of variation from $HSR_{sim}$ across wavelengths in different data sets (see Sect. "Accuracy of PROSPECT model across wavelengths" and Fig. 2), the high-error wavebands are expected to play a crucial role in any $HSR_{meas}$-$P_{inv}$ model that aims to achieve a transferability performance close to that of the $HSR_{sim}$-$P_{inv}$ models. More specifically, an ideal model is expected to minimize the weights of the features corresponding to inaccurate wavelength regions during training.

**Fig. 6:**
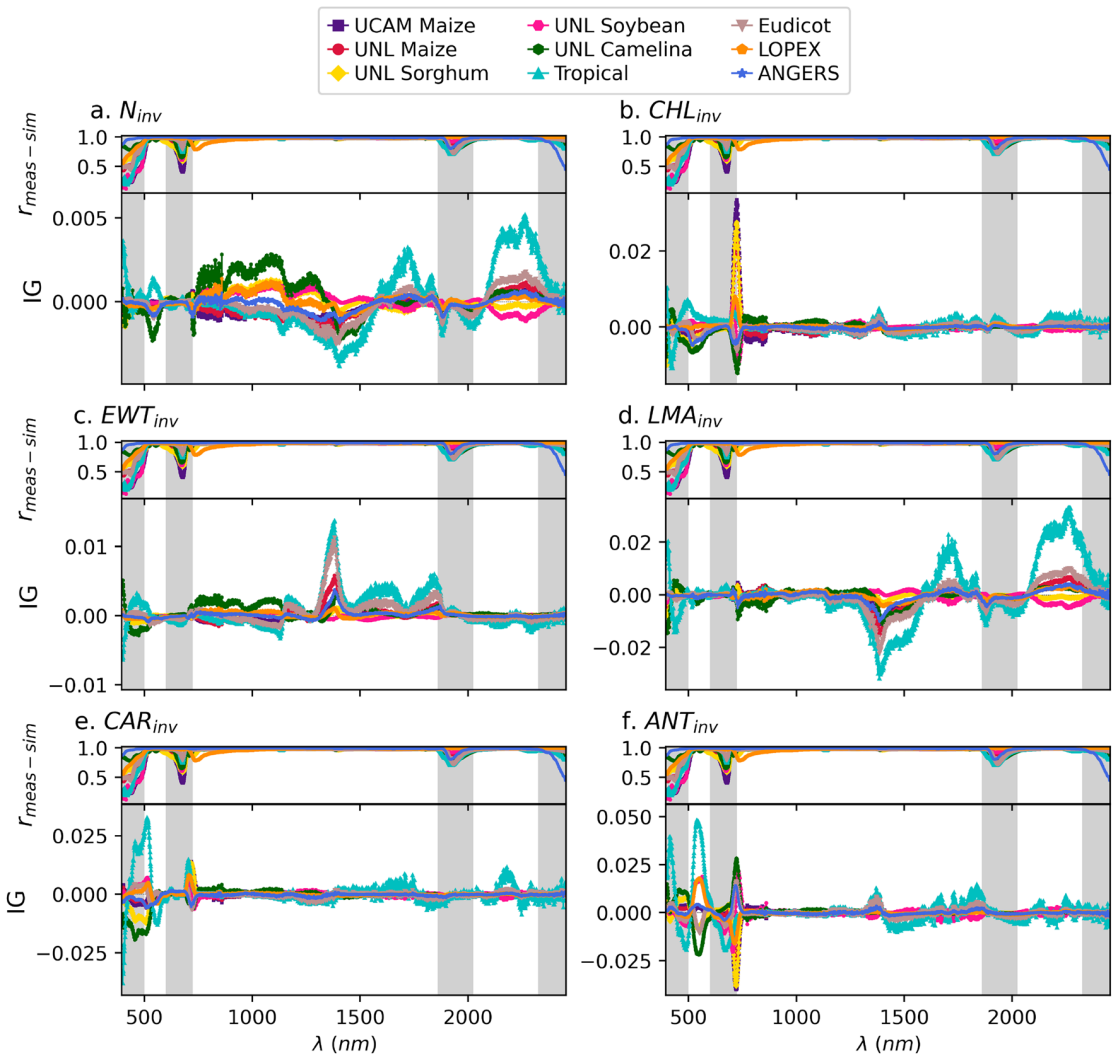
Relation between feature importance from neural network (NN) and PROSPECT inversion errors. The average integrated gradient (IG) scores of features, *i.e.*, reflectance values at given wavelengths, for separate NN models that predict (**a**) leaf structure parameter ($N_{inv}$), (**b**) chlorophyll content ($CHL_{inv}$), (**c**) equivalent water thickness ($EWT_{inv}$), (**d**) leaf mass per area ($LMA_{inv}$), (**e**) carotenoid content ($CAR_{inv}$), and (**f**) anthocyanin content ($ANT_{inv}$). For all traits, IG were computed on nine plant species data sets, UCAM Maize, UNL Maize, UNL Sorghum, UNL Camelina, UNL Soybean, ANGERS, Eudicot, Tropical, and LOPEX, using a model trained on the LOPEX data set. The upper panels show the Pearson correlation coefficient ($r_{meas\text {-}sim}$) between the $HSR_{meas}$ and $HSR_{sim}$ samples across the same data sets. Shaded regions, marked in gray, indicate wavelength ranges with low accuracy.

To assess the contribution of each feature (*i.e.*, wavelength) in the trained neural network models, we computed the average Integrated Gradients (IG) across samples from all data sets based on the model trained on the LOPEX data set. This selection was based on the performance of the models trained on this data set, which showed the highest transferability among the data sets analyzed. Integrated Gradients is a recent attribution technique that has been widely used for interpreting deep learning models in terms of the contribution of each input feature to the model’s prediction^63^. Interestingly, this importance analysis showed notably small IG values for the high-error wavebands across the models corresponding to the four highly transferable inversion traits (Fig. 6a,b). This finding indicates that the configuration of our neural network models successfully minimizes the contributions of inaccurate regions, while attributing the main importance to the wavelength ranges where the PROSPECT model performs well. On the other hand, we observed slight overlaps between the wavebands with high IG values and those with high error for $CAR_{inv}$ and $ANT_{inv}$ (Fig. 6c,d). This, in turn, explains the lower transferability performance of the $HSR_{meas}$-$P_{inv}$ models (Fig. S5e,f,k,l) compared to the corresponding $HSR_{sim}$-$P_{inv}$ models (Fig. 4e,f,k,l) for these traits.

### A fast transferable framework for ML-based inversion of measured hyperspectral reflectance data

Beyond its accuracy, the PROSPECT inversion model (*i.e.*, estimating $P_{inv}$ from $HSR_{meas}$ data) involves a notably higher computational cost compared to the forward mode (*i.e.*, simulating the $HSR_{sim}$ profile from input parameters), primarily due to the optimization process. To contribute a faster inversion approach, we developed a global neural network model with the same configuration as the models described in Sect. "Neural network models robustly maintain the transferability of PROSPECT inversion model across plant species", using LOPEX and ANGERS as training data sets. These data sets were selected based on their strong performance in predicting inversion parameters from $HSR_{meas}$ profiles (Fig. S5; Sect. "Description of the experimental data sets"). In addition, their high species diversity and prior use in calibrating PROSPECT models^34,35,38^ suggest that the trained model can be robustly applied to species data sets beyond those analyzed. To compare the performance of our ML-based inversion model with the forward PROSPECT model, we also trained an equivalent PLSR model on the $HSR_{sim}$-$P_{inv}$ pairs from the same data sets.

**Fig. 7:**
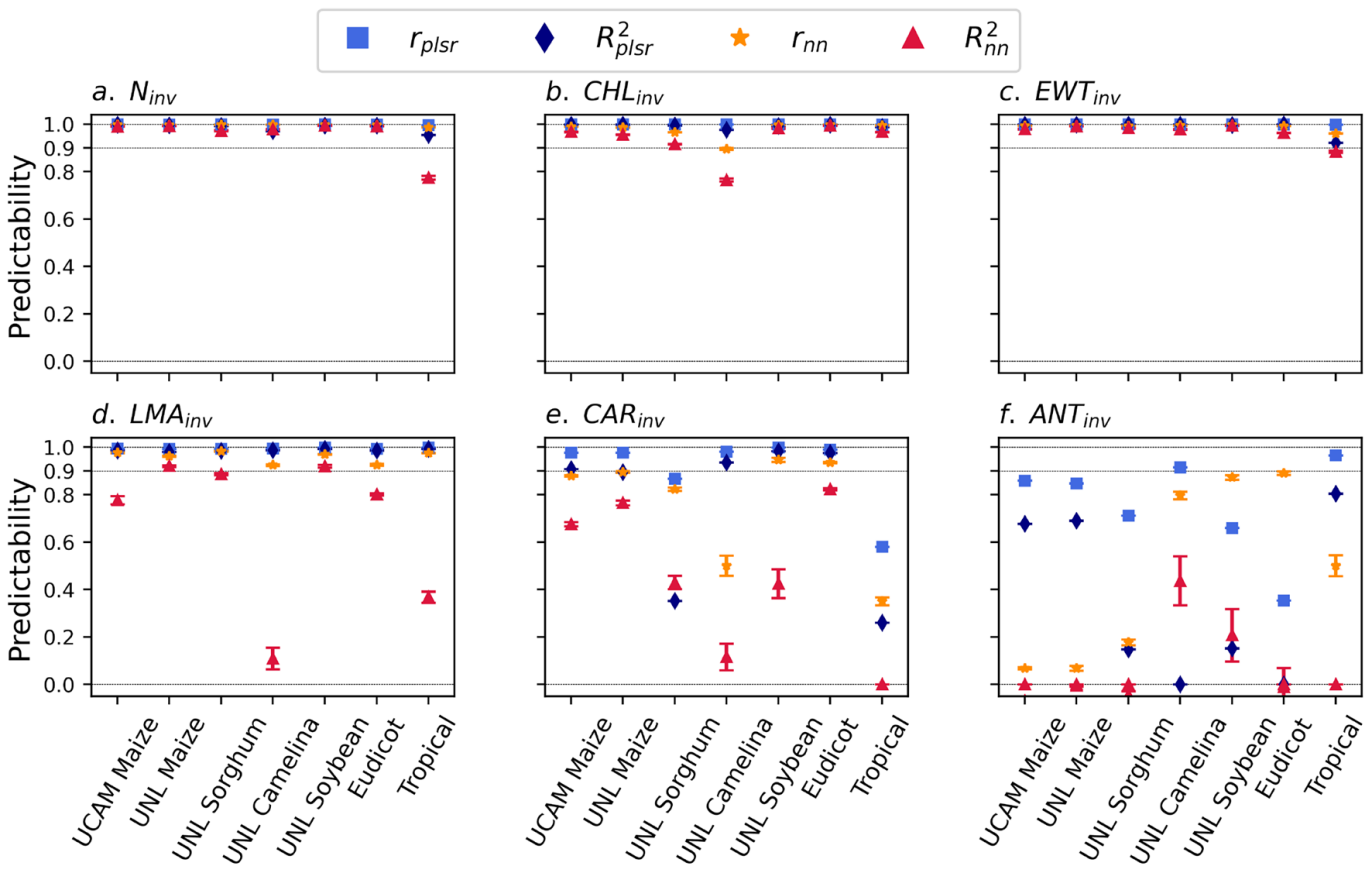
Predictive performance of models trained on diverse data sets. The predictability scores of PROSPECT inversion traits ($P_{inv}$) were evaluated using measured ($HSR_{meas}$) and simulated ($HSR_{sim}$) hyperspectral reflectance data, based on neural network (NN) and partial least squares regression (PLSR) models, respectively. The models were trained on the LOPEX and ANGERS data sets and tested on seven species data sets: UCAM Maize, UNL Maize, UNL Sorghum, UNL Camelina, UNL Soybean, Eudicot, and Tropical. All the predictability scores indicate the ensemble average over 10 NN models with random initial configurations. The errors indicate the mean standard deviation of the scores over ensemble. The predictability values of $R^2=0$ and $r=0$ indicate either the exact value of 0 or smaller values.

Interestingly, our global NN model showed excellent predictive performance (*i.e.*, $r \rightarrow 1$ and $R^2 \rightarrow 1$), with only a few exceptions, for the three inversion parameters $N_{inv}$, $CHL_{inv}$, and $EWT_{inv}$ across all seven species data sets: UCAM Maize, UNL Maize, UNL Sorghum, UNL Camelina, UNL Soybean, Eudicot, and Tropical (Fig. 7a–c). These performances were also significantly higher than those of the models trained individually on each of these data sets, especially in terms of $R^2$ (Fig. S5a–c,g–i and Fig. 7a–c). On the other hand, the exceptional cases (*e.g.*, $N_{inv}$ on the Tropical data set) still exhibited excellent *r* values and relatively high $R^2$ values. Importantly, these predictability values were notably close to those of the equivalent forward-based PLSR model, indicating the ability of our ML-based inversion approach in preserving the predictability of the PROSPECT forward model. For $LMA_{inv}$, we observed similar trends in *r* scores for our global NN model, while the $R^2$ values were notably smaller than those of the PLSR model for the UNL Camelina and Tropical data sets (Fig. 7d). This finding also indicated the success of our ML-based inversion approach in capturing the trends for these traits based on $HSR_{meas}$. In contrast, our global NN approach showed weak predictive performance for the two traits, $CAR_{inv}$ and $ANT_{inv}$ (Fig. 7e,f), in the majority of models. However, the forward-based PLSR model exhibited similarly low performance, indicating the mechanistic origin of these results as discussed in Sect. "Sensitivity analysis explains the transferability patterns of the PROSPECT-D model in the forward mode".

## Discussion

Whilst PROSPECT models offer a robust mechanistic framework for simulating leaf optical reflectance and transmittance spectra across different wavelengths, developing data-based strategies to improve these models and their real-world use in obtaining information about leaf traits from HSR data remains essential. To this end, assessment the error patterns of the model across diverse data sets and species is key to their wider applications. At the same time, developing transferable surrogate data-driven models that preserve the mechanistic basis of the original models while reducing computational cost is another important aspect of widening the applications.

### An innovative design for assessment of the errors of PROSPECT-D model

In the context of extending the PROSPECT model’s utility, a comprehensive understanding of its errors across wavelengths of HSR profiles (*i.e.*, the differences between measured and simulated spectra) on a broad range of data sets is crucial for assessing the model generalization. To perform such analysis, each measured HSR profile must be paired with a corresponding set of leaf biological traits, *e.g.*, *N* and *EWT* (see Sect. "PROSPECT-D model implementation"), derived from the same sample material as the HSR data. These traits serve as input parameters to the PROSPECT model, enabling the simulation of HSR profiles and facilitating direct comparison between the simulated and experimental spectra, as well as further error analyses.

Due to the difficulty of measuring the input traits of the PROSPECT models, the majority of previously published data sets for leaf HSR do not include corresponding sets of those input parameters. As a result, comprehensive evaluations of the PROSPECT model’s accuracy across different plant species and measurement conditions remain limited (see Sect. "Introduction"). To address this concern, in this work, we employed a different approach by combining the PROSPECT inversion and forward modes as a synthetic data re-generation cycle (see Sect. "Synthetic data generation" and Fig. 1). More specifically, for each measured HSR profile, $HSR_{meas}$, we estimated the corresponding input parameters, $P_{inv}$, using the PROSPECT inversion model, and then simulated a corresponding HSR profile (*i.e.*, $HSR_{sim}$) from the resulting parameters. This strategy enabled us to analyze the errors of the spectra simulated by PROSPECT across different wavelengths for all data sets using only the measured HSR profiles, without requiring any measured input parameters.

Based on this analysis, the PROSPECT-D model demonstrated high accuracy for simulating HSR profiles, in terms of Pearson correlation and relative error, for all data sets across nearly all wavelengths. Interestingly, the only exceptions were four narrow wavebands, which correspond to spectral regions exhibiting very low reflectance values (Sect. "Accuracy of PROSPECT model across wavelengths" and Fig. 2a,b). Consistent with this observation, we demonstrated that the reflectance values at individual wavelengths, treated as effective traits, in these inaccurate wavebands exhibited low broad-sense heritability across samples of different genotypes and conditions, in comparison to other spectral regions (Sect. "Environmental effects explain the errors of the PROSPECT-D model" and Fig. 3). This indicated that the environmental factors had a notably stronger influence than genetic variation in this regions, during the measurement of HSR profiles. This raises the question of whether more reliable reflectance values result from actual measurements or from simulations by the PROSPECT models, a question that can be investigated in future work.

### The relevance of inversion-based error analysis

The relevance of our error assessment approach strongly depends on the accuracy of the PROSPECT inversion model. Specifically, an ideal inversion model must thoroughly explore the parameter space and identify the optimal set of input values that minimize discrepancies between observed and simulated HSR profiles. Therefore, when the inversion model successfully estimates these optimal parameters, the inversion-based approach not only becomes highly relevant for error analysis, but it also avoids additional biases possibly incurred by measured parameters. Such biases may arise from measurement errors or mismatches in environmental conditions between the HSR acquisition and leaf trait assessment.

To evaluate the accuracy of the employed PROSPECT-D inversion approach (see^48^), we extended the data re-generation process for the simulated HSR profiles ($HSR_{sim}$). Namely, we re-estimated the PROSPECT input parameters ($P_{re}$) from $HSR_{sim}$, then used them to re-simulate the HSR spectra ($HSR_{re}$) (see Sect. "Synthetic data generation" and Fig. 1). Interestingly, we observed no errors between $HSR_{sim}$ and $HSR_{re}$ at any wavelength (Sect. "Accuracy of PROSPECT model across wavelengths" and Fig. 2a,b). This indicated that when the HSR profiles fall within the domain recognized by the PROSPECT model, the inversion process can estimate the input parameters with perfect accuracy. This provides evidence for the robustness of the inversion model and, in turn, supports the relevance of our approach.

### Transferability of the machine learning models trained on the PROSPECT-D forward model across data sets

Considering the observed errors induced by the PROSPECT-D model, we then asked whether this model robustly captures the species-specific variations of the HSR profiles based on the variations in the corresponding input parameters across species data sets. Towards a data-driven surrogate approach for PROSPECT-D model, we proposed a novel ML-based framework that considers the transferability of the models across data sets from different species. Namely, we investigated the relationships between input parameters from various data sets and their corresponding simulated HSR profiles. To do this, we tested whether and to what extent the relationship between $P_{inv}$ and $HSR_{sim}$ in one data set could be predicted using a model trained on another. Specifically, we built ML models using PLSR, treating $HSR_{sim}$ as feature data and $P_{inv}$ as response data. Each model was trained on one of the nine species-specific data sets and evaluated on all others as test data sets (see Sect. "The PROSPECT-D forward model shows excellent transferability across plant species"). This idea, indeed, provided a data-based approach to examine the transferability of the PROSPECT-D forward model across plant species.

This analysis demonstrated excellent predictive performance, in terms of *r* and $R^2$ scores, for four traits (*i.e.*, PROSPECT input parameters), namely, $N_{inv}$, $CHL_{inv}$, $EWT_{inv}$, and $LMA_{inv}$, for the majority of models, with a few exceptions. Notable exceptions occurred when the models were tested on data sets with more diverse species (*e.g.*, ANGERS and LOPEX) than those present in the training data (Fig. 4a–d, g–j). Specifically, we observed that differences in the distributions of the test and target data sets play a more important role than sample size. For example, we found that a model trained using the UNL Camelina data set comprising 96 samples could successfully predict the *N* parameter on the UCAM Maize data set comprising 3749 samples, but failed on the ANGERS and LOPEX data sets comprising 308 and 330 samples, respectively (see Fig. 4 and Fig. S3).

In contrast, we observed considerably weaker transferability between data sets for the two traits $CAR_{inv}$ and $ANT_{inv}$ (Fig. 4e,f,k,l). To have a data-based explanation for the poor performance of these two traits, we preformed a sensitivity analysis on the HSR profiles (see Sect. "Sensitivity analysis explains the transferability patterns of the PROSPECT-D model in the forward mode"). This analysis revealed a low sensitivity for PROSPECT-D model in a narrow waveband for both traits for all data sets (Fig. 5e,f). At the same time, these two traits also showed generally long-tailed distributions compared those of other traits across the analyzed data sets (Fig. S3e,f). These suggested that the model suffers from a non-identifiability problem for this traits, *i.e.*, different values of the parameters often result in highly similar HSR profiles^61^. This limited sensitivity can be explained by the spectral absorption characteristics of carotenoids and anthocyanins, which, as pigments, are masked by chlorophyll in their spectral region. As a result, they exhibit overlapping absorption features, reducing their independent detectability (see^35^ and references therein).

### Transferability of the machine learning models trained on the PROSPECT-D inversion model across data sets

Based on the observed ML-based transferability patterns in the ML models trained on the PROSPECT forward mode, provided by $HSR_{sim}$-$P_{inv}$ PLSR models, we then asked whether similar transferability patterns exist across species in the relationship between $HSR_{meas}$ and $P_{inv}$. Compared to earlier scenarios, the $HSR_{meas}$-$P_{inv}$ models are more relevant in real-world applications, as they offer a practical way to extract leaf trait information directly from HSR profiles.

In this regard, for each $HSR_{sim}$-$P_{inv}$ PLSR model, we trained an equivalent $HSR_{meas}$-$P_{inv}$ NN model that showed comparably high transferability between different data sets (Fig. S5) to the corresponding PLSR models (Fig. 4) (see Sect. "Neural network models robustly maintain the transferability of PROSPECT inversion model across plant species"). We then investigated the feature importance of the NN models, using integrated gradients (IG), allowing us to identify the wavelengths that more significantly contribute to the predictions of the NN models (see Sect. "Feature importance of neural network models explains the high transferability of PROSPECT inversion models"). This analysis revealed that for the four high-performance traits, $N_{inv}$, $CHL_{inv}$, $EWT_{inv}$, and $LMA_{inv}$, the wavelengths associated with higher prediction errors had very low IG values (Fig. 6). In other words, our neural network models do not use the wavebands with high error from simulations.

### ML-based surrogate model for PROSPECT-D inversion

Despite the importance of the inversion of the PROSPECT model in extracting information about leaf traits from HSR data, it relies on an iterative optimization process. Therefore, the inversion of the PROSPECT model becomes significantly more computationally intensive than its forward mode. This highlights the need for developing surrogate models for the PROSPECT suite of models. In this context, the strong performance of the neural network models suggested their potential for predicting PROSPECT inversion parameters. Here, we demonstrated that a NN model trained on the measured HSR profiles of the two diverse data sets, *i.e.*, ANGERS and LOPEX, can reliably predict the four PROSPECT-D inversion parameters, $N_{inv}$, $CHL_{inv}$, $LMA_{inv}$, and $EWT_{inv}$, across most of the other data sets. This result establishes a robust framework for efficiently and rapidly estimating leaf traits from HSR profiles.

To further improve the NN model computational efficiency, we also trained multi-target neural network models, each configured to predict different combinations of traits as joint output variables. We observed that these configurations can also lead to generally high predictive performance. For example, the models trained on the LOPEX and ANGERS data sets with three output traits, $N_{inv}$, $CHL_{inv}$, and $EWT_{inv}$, provided equally high results for each of these traits over all remaining data sets (see Fig. S6 and Table S1).

Although the NN models showed strong transferability across the evaluated data sets, their generalization toward a universal model across most plant species remains challenging due to limitations in training data size and the diversity of species and environmental conditions. In particular, improved performance would require more refinement for $CAR_{inv}$ and $ANT_{inv}$, which are more sensitive to these limitations due to the inherent noise associated with the mechanistic model.

In addition to the general model based on the diverse data sets, our ML-based approach can also be adapted to subsets of a data set to predict traits in the remaining samples or across data sets with similar domain spaces. For example, we demonstrated that our NN models are strongly transferable between the UCAM Maize and UNL Maize data sets for $N_{inv}$, $CHL_{inv}$, $EWT_{inv}$, and $LMA_{inv}$, as well as for $CAR_{inv}$ (Fig. S5). A more detailed investigation of local transferability patterns is proposed for future work.

## Conclusion

In summary, we provided a data-driven approach to analyze the performance and transferability of the ML-based models trained on top of the PROSPECT-D model across a large set of measured HSR data sets. Our results provide not only an efficient inversion framework for retrieving information about leaf traits from measured HSR profiles, but also new utilities for the computational and theoretical improvement of the PROSPECT model. First, our framework extends the error analysis for any HSR data without requiring corresponding measurements of leaf biochemical or structural traits. This promises the capacity to detect model limitations beyond small data sets by extending the analysis to large, publicly available data from diverse plant species and measurement conditions.

In line with this, our heritability analysis provides a general insight into how model accuracy is influenced by environmental and genetic factors. However, we recommend the deeper assessment of the structural assumptions in the PROSPECT model, such as simplified representations of leaf structure^37,64^, as additional potential sources of errors in the transferability of the ML models to be explored in future work.

Our transferability analysis further demonstrates the patterns of the relationships between HSR spectra and leaf traits across species and growth conditions. While the transferability of the ML models is not a direct proxy for the transferability of the PROSPECT model itself, it can still provide useful insights. In particular, the observed transferability of the ML models may partially reflect that of PROSPECT under its current calibration, as the ML emulators capture the underlying information flow across data sets. Along this line, these transferability patterns can also help highlight the most appropriate data sets for calibration of the PROSPECT model by identifying those that show the highest performance. Finally, our data-driven approach does not explicitly incorporate the physics principles underlying the mechanistic model. Instead, it relies on surrogate ML models for its inversion and forward modes. This allows the application of the surrogate models in the assessment of other radiative transfer models in addition to the PROSPECT family of models.

## Supplementary Information

Supplementary Information.

## Data Availability

The UNL (Maize, Sorghum, Camelina, and Soybean) data sets are available upon request from the corresponding author^5^. The UCAM Maize^25,44^, LOPEX^47^, ANGERS^34,35,48^, Eudicot^46^, and Tropical^45^ data sets are already publicly available. The Python and R codes used for our analysis are available on the following URL: github.com/MRahimiMajd/prospect_transferability.
