# Supplementary material for: Machine learning surrogate for the leaf PROSPECT-D model and its applications across plant species
Source: Sci Rep. 2026 May 19;16:15602. doi: 10.1038/s41598-026-53899-1 (PMC13187411; doi:10.1038/s41598-026-53899-1)
Supplement: Supplementary file 1 — Supplementary Information. [file 41598_2026_53899_MOESM1_ESM.pdf]

Supplementary materials for ”Machine learning surrogate for the leaf PROSPECT-D model and its applications across plant species”

Milad Rahimi-Majd[1,2], Rudan Xu[1,2], Stefan Bauermeister[1], and Zoran Nikoloski* [1,2]

[1]Bioinformatics Department, Institute of Biochemistry and Biology, University of Potsdam, 14476 Potsdam, Germany
[2]Systems Biology and Mathematical Modeling Group, Max Planck Institute of Molecular Plant Physiology, 14476 Potsdam, Germany

*Contact: nikoloski@mpimp-golm.mpg.de

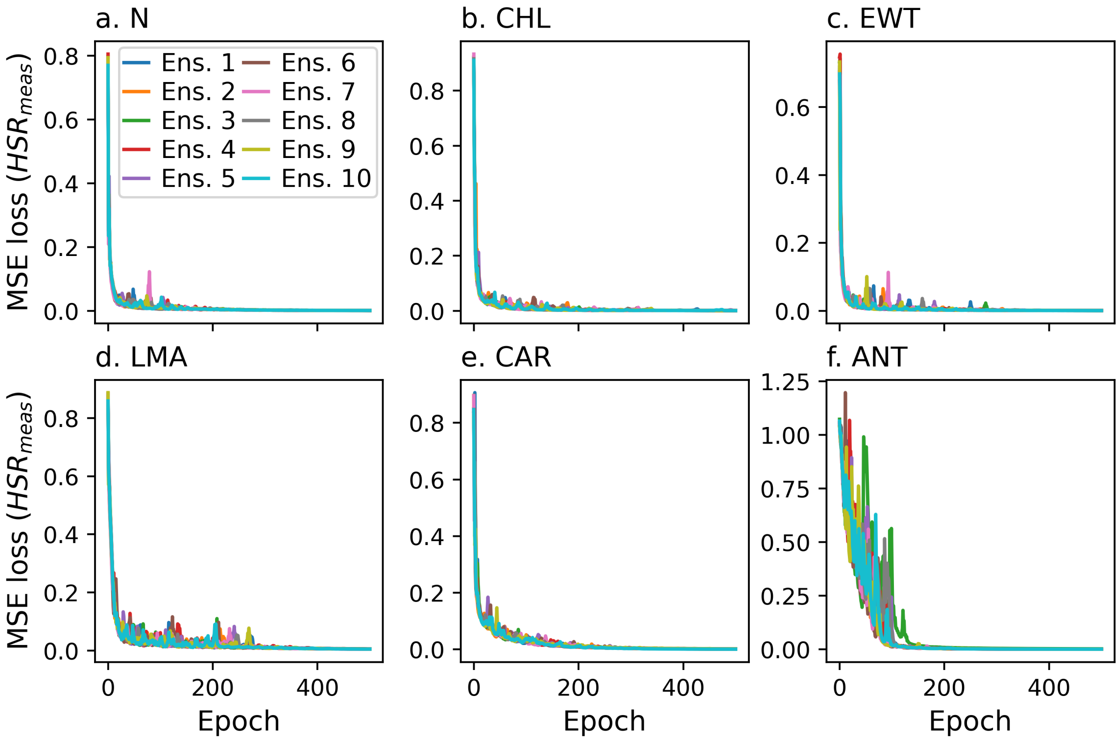

Figure S1: Evaluation of loss function of neural networks. The loss decay, over epochs, during training NN models on ANGERS and LOPEX data sets with target variables of (a) N_{inv}, CHL_{inv}, and EWT_{inv}, (b) LAM_{inv}, and (c) CAR_{inv} for HSR_{meas}-P_{inv} training scenarios. (d)-(f) The same with (a)-(c) respectively, for HSR_{sim}-P_{inv} training scenarios.

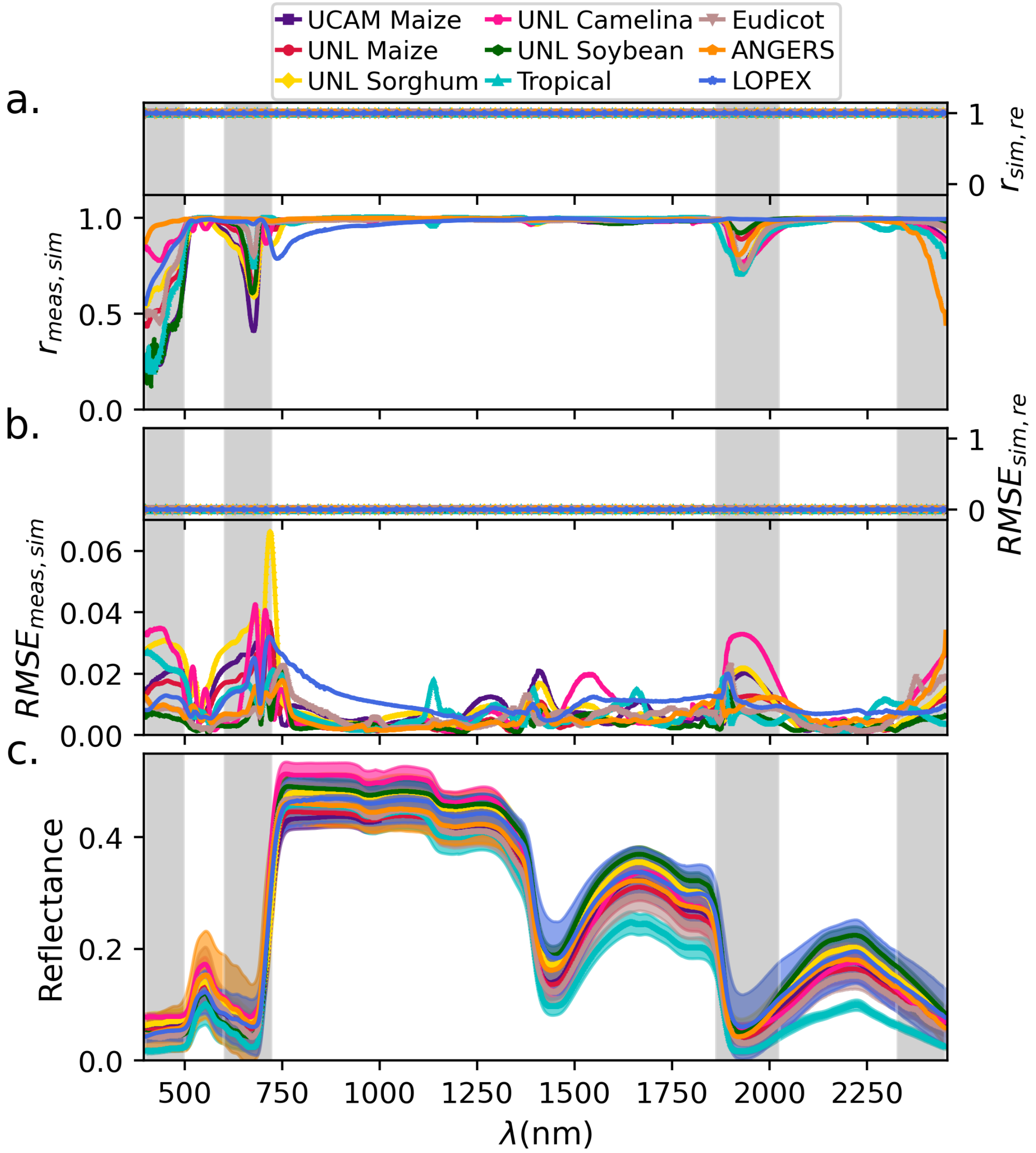

Figure S2: Accuracy of PROSPECT model across wavelengths based on root mean square error (RMSE). (a) Pearson correlation ($r_{meas,sim}$) and (b) RMSE ($RMSE_{meas,sim}$) between measured (HSR_{meas}) and simulated (HSR_{sim}) leaf hyperspectral reflectance across wavelengths from 400 to 2450 nm, for data sets of nine species: UCAM Maize, UNL Maize, UNL Sorghum, UNL Camelina, UNL Soybean, ANGERS, LOPEX, Eudicot, and Tropical. To enhance the visual representation of the variation, the upper bound of the y-axis in panel (b) was set to 1. Insets in panels (a) and (b) show the corresponding values—$r_{sim,re}$ and $RMSE_{sim,re}$—between the simulated (HSR_{sim}) and re-simulated (HSR_{re}) reflectance for the same data sets. (c) The average of HSR_{meas} samples across wavelengths for the analyzed data sets, with shaded regions in matching colors representing the corresponding standard deviations.

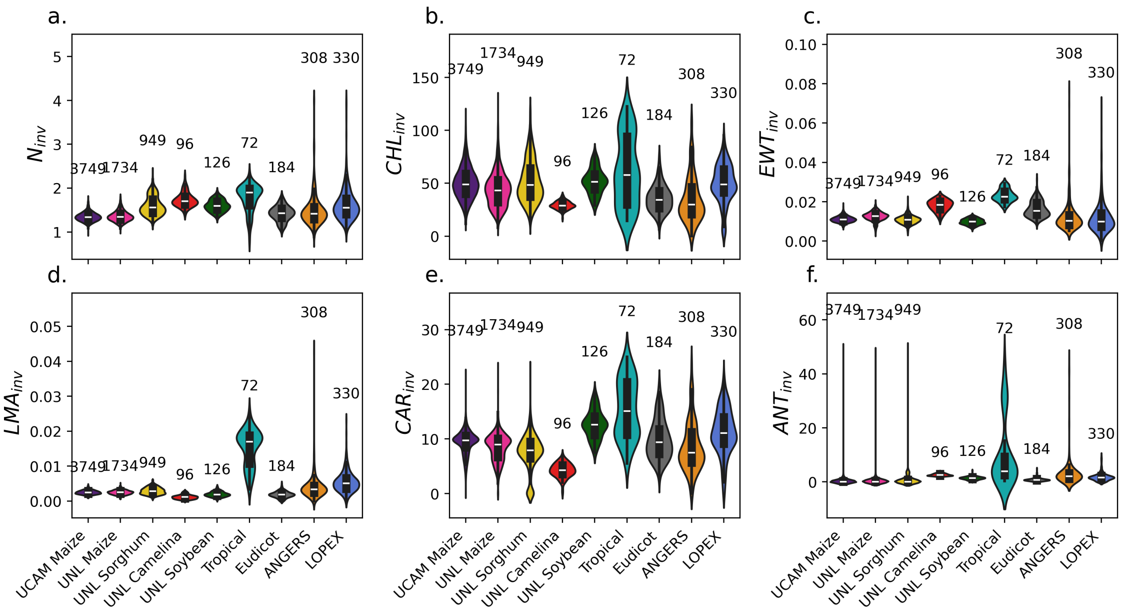

Figure S3: The distribution of PROSPECT inversion parameters. Violin plots of the PROSPECT inversion parameters, (a) leaf structure parameter (N_{inv}), (b) chlorophyll content (CHL_{inv}), (c) equivalent water thickness (EWT_{inv}), (d) leaf mass per area (LMA_{inv}), (e) carotenoid content (CAR_{inv}), and (f) anthocyanin content (ANT_{inv}), for nine species datasets: UCAM Maize, UNL Maize, UNL Sorghum, UNL Camelina, UNL Soybean, ANGERS, LOPEX, Eudicot, and Tropical. The numbers above the violin plots show the sample size for each distribution.

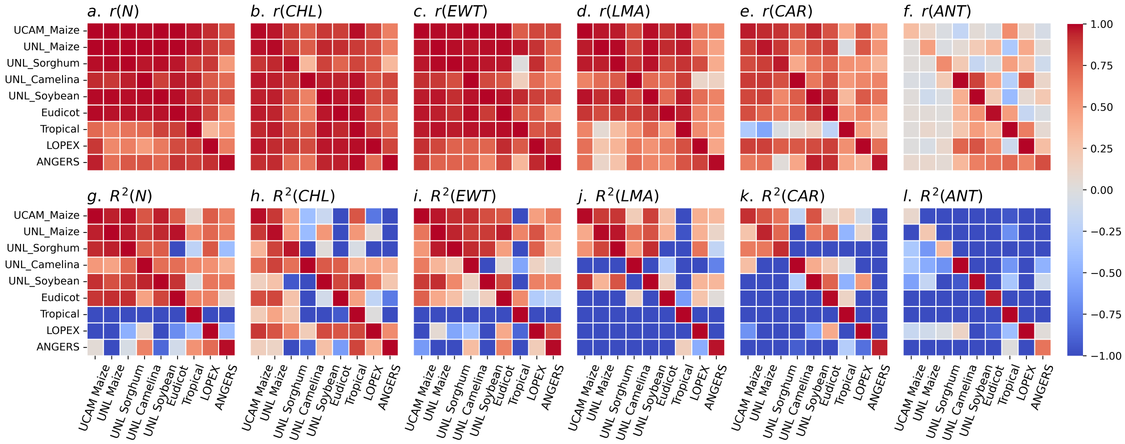

Figure S4: Transferability of PROSPECT-D inversion mode based on partial least squares regression (PLSR) models across plant species. (a)-(f) The heatmap plots of pairwise Pearson correlation (r) predictability of prospect inversion parameters, P_{inv}, *i.e.*, leaf structure parameter (N_{inv}), chlorophyll content (CHL_{inv}), equivalent water thickness (EWT_{inv}), leaf mass per area (LMA_{inv}), carotenoid content (CAR_{inv}), and anthocyanin content (ANT_{inv}), respectively, through the trained models on their corresponding measured hyperspectral reflectance (HSR_{meas}) profiles. The rows and columns of the heatmap plots indicate the train and test data sets, respectively. (g)-(l). The same as with (a)-(f), respectively, with the predictability score corresponding to the coefficient of variation R^2. To retain the same scale, the values of $R^2 < -1$ are denoted by -1. All the trained models are based on PLSR with 30 components.

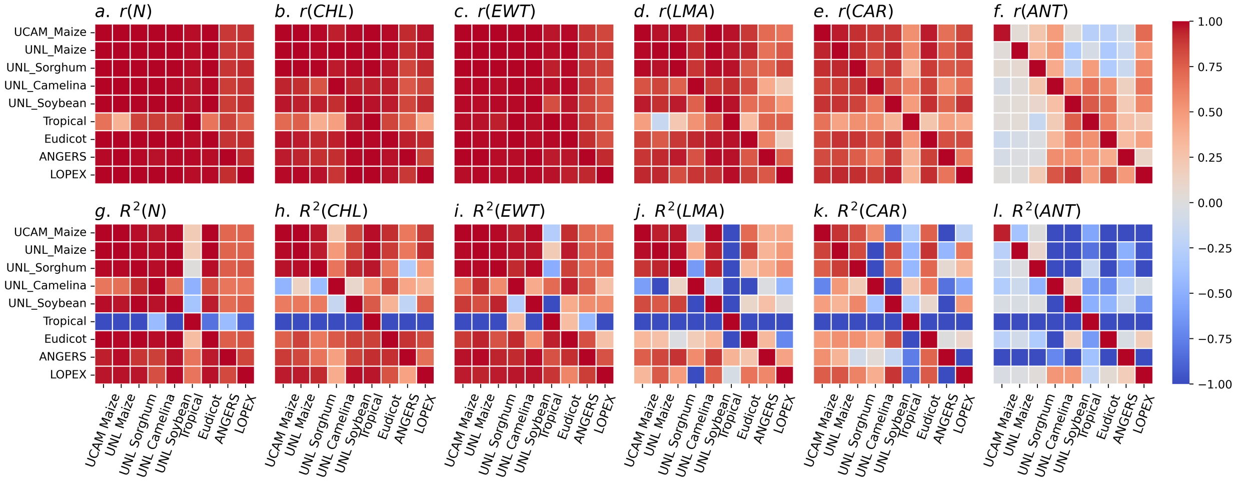

Figure S5: Transferability of PROSPECT-D inversion mode based on neural network (NN) models across plant species. (a)-(f) The heatmap plots of pairwise Pearson correlation (r) predictability of prospect inversion parameters, P_{inv}, *i.e.*, leaf structure parameter (N_{inv}), chlorophyll content (CHL_{inv}), equivalent water thickness (EWT_{inv}), leaf mass per area (LMA_{inv}), carotenoid content (CAR_{inv}), and anthocyanin content (ANT_{inv}), respectively, through the trained models on their corresponding measured hyperspectral reflectance (HSR_{meas}) profiles. The rows and columns of the heatmap plots indicate the train and test data sets, respectively. (g)-(l). The same as with (a)-(f), respectively, with the predictability score corresponding to the coefficient of variation R^2. To retain the same scale, the values of $R^2 < -1$ are denoted by -1. For each training data sets, six neural network (NN) models with individual target variables of N_{inv}, CHL_{inv}, EWT_{inv}, LMA_{inv}, CAR_{inv}, and ANT_{inv} were trained. All the predictability scores indicate the ensemble average over 10 NN models with random initial configurations.

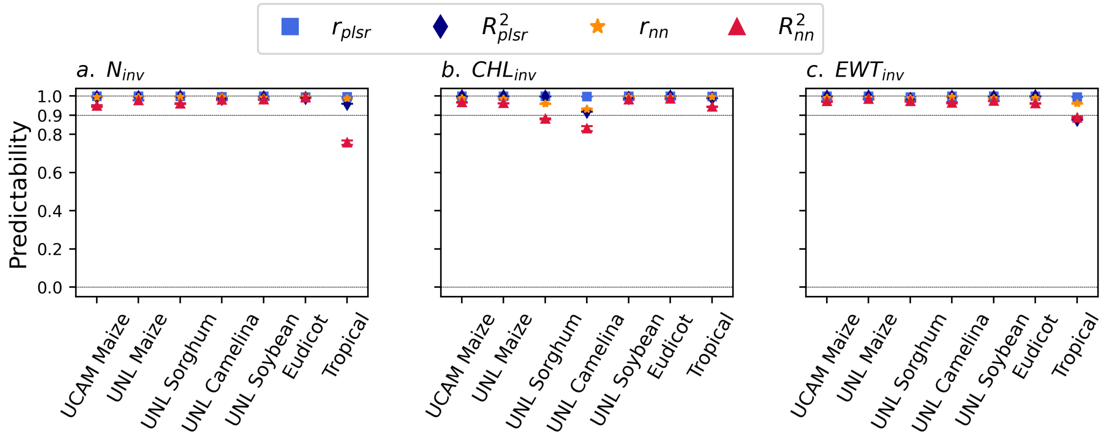

Figure S6: Predictive performance of multi-target models trained on diverse data sets. The predictability scores of PROSPECT inversion traits (P_{inv}) were evaluated using measured (HSR_{meas}) and simulated (HSR_{sim}) hyperspectral reflectance data, based on neural network (NN) and partial least squares regression (PLSR) models, respectively. The NN models were jointly trained for the three parameters, N_{inv}, CHL_{inv}, and EWT_{inv} as target variables. The models were trained on the LOPEX and ANGERS data sets and tested on seven species data sets: UCAM Maize, UNL Maize, UNL Sorghum, UNL Camelina, UNL Soybean, Eudicot, and Tropical. All the predictability scores indicate the ensemble average over 10 NN models with random initial configurations. The errors indicate the mean standard deviation of the scores over ensemble.

Table S1: Comparing the predictive performance of neural network models in single- and multi-target scenarios. Comparison of single-target and multi-target neural network (NN) model performance on the PROSPECT-D inversion traits N_{inv}, CHL_{inv}, and EWT_{inv}. The comparison is based on the difference in the coefficient of determination (R^2_{nn}), defined as $\Delta R^2_{nn} = R^2_{nn}(multi) - R^2_{nn}(single)$. The models were trained on the LOPEX and ANGERS data sets and tested on data sets from seven species: UCAM Maize, UNL Maize, UNL Sorghum, UNL Camelina, UNL Soybean, Eudicot, and Tropical. All the predictability scores indicate the ensemble average over 10 NN models with random initial configurations. The errors indicate the mean standard deviation of the scores over the ensemble.

Test data set	Trait	$R^2_{nn}(single)$	$R^2_{nn}(multi)$	ΔR^2_{nn}
Eudicot	CHL_{inv}	0.994 ± 0.000	0.986 ± 0.001	-0.008
Eudicot	EWT_{inv}	0.963 ± 0.001	0.962 ± 0.001	-0.001
Eudicot	N_{inv}	0.991 ± 0.001	0.992 ± 0.001	+0.001
Tropical	CHL_{inv}	0.967 ± 0.001	0.944 ± 0.002	-0.023
Tropical	EWT_{inv}	0.884 ± 0.004	0.887 ± 0.006	+0.003
Tropical	N_{inv}	0.775 ± 0.008	0.757 ± 0.011	-0.018
UCAM Maize	CHL_{inv}	0.968 ± 0.002	0.967 ± 0.001	-0.001
UCAM Maize	EWT_{inv}	0.980 ± 0.001	0.973 ± 0.003	-0.007
UCAM Maize	N_{inv}	0.990 ± 0.001	0.948 ± 0.005	-0.042
UNL Camelina	CHL_{inv}	0.765 ± 0.007	0.830 ± 0.012	+0.065
UNL Camelina	EWT_{inv}	0.978 ± 0.001	0.965 ± 0.004	-0.013
UNL Camelina	N_{inv}	0.978 ± 0.002	0.981 ± 0.001	+0.003
UNL Maize	CHL_{inv}	0.956 ± 0.001	0.963 ± 0.001	+0.007
UNL Maize	EWT_{inv}	0.991 ± 0.000	0.984 ± 0.001	-0.007
UNL Maize	N_{inv}	0.993 ± 0.000	0.978 ± 0.001	-0.015
UNL Sorghum	CHL_{inv}	0.915 ± 0.001	0.880 ± 0.003	-0.035
UNL Sorghum	EWT_{inv}	0.983 ± 0.001	0.974 ± 0.003	-0.009
UNL Sorghum	N_{inv}	0.973 ± 0.002	0.961 ± 0.002	-0.012
UNL Soybean	CHL_{inv}	0.982 ± 0.001	0.982 ± 0.001	0.000
UNL Soybean	EWT_{inv}	0.994 ± 0.000	0.975 ± 0.002	-0.019
UNL Soybean	N_{inv}	0.994 ± 0.001	0.981 ± 0.001	-0.013
